# Sectional Medicine

**Published:** 1848-10

**Authors:** 


### THE MEDICAL EXAMINER.

### PHILADELPHIA, OCTOBER, 1848.

### SECTIONAL MEDICINE.

The last number of the Southern Medical and Surgical Journal, (Sept., 1848,) under the head of "*Medical College Circulars,*" contains some editorial remarks, which, after other articles that have appeared in recent numbers, pains, more than surprises, us. It would seem as if Jefferson Medical College had become an object of especial dislike to the learned editor, and, in the paragraph to which we have referred, he—by implication, at least—charges charlatanism upon the whole body of professors.

After some general observations relating to College Circulars, the

Medical Convention, extension of the lecture term, &c., the following remarks occur:

"As a Southerner, we cannot pass without comment the second paragraph in the Annual Announcement of the Jefferson Medical College, because it is evidently a renewal of the attempt made in the same and other quarters to undervalue local advantages which we feel that we, in common with our Southern and South-western brethren, undoubtedly possess in the study and treatment of Malarious diseases. The passage is as follows:

"'The idea that a student of Medicine must be taught his profession in the very locality in which he is destined to practice it, is now generally, as it ought to be, universally, abandoned. It must be admitted that the great principles of the Science are the same every where, and that the student ought for his own sake, [for the sake of the Jefferson Medical College?—Ed.] to seek for information wherever it can be best and most readily obtained.'"

Did not the Editor's cheek burn when he penned the words in brackets, introduced by him in the above quotation? To recommend to students "to seek for information wherever it can be best and most readily obtained," means to direct them to Jefferson Medical College! Well, if that be the true interpretation, who has a right to complain? But did it not occur to our Southern champion, that if by the inculcation of such sentiments the professors of Jefferson Medical College render themselves obnoxious to the charge of charlatanry, and a wish to attract students to their school, he, in advocating the contrary doctrine, might be suspected of a wish to detain them at the "Georgia Medical College," in Augusta? We, however, make no such allegation. Much as we are inclined to follow his example on other occasions, we will not imitate it on this. It is charged that an attempt is made to undervalue the local advantages of the South in the study and treatment of malarious diseases! The obnoxious paragraph in the "Annual Announcement" is cited, and yet it contains not a word of the kind. What right has he to infer that the malarious diseases of the South are alluded to, any more than the milk-sickness of the West, or the pleurisies, pneumonias and adynamic fevers of the North? Could not the East and the West, and the North, make the same charge with equal propriety? Undoubtedly.

"Pulmonary affections," says the Richmond College Circular, "are as prevalent at the North, as intermittent and remittent fevers in the Southern Atlantic States, and hepatic affections in warmer regions." Therefore, they who are educated at Richmond and Augusta must be incompetent to treat a pneumonia or a hepatitis?

It might be thought from the remarks we have quoted that no one can know any thing about malarious diseases but those who live in the South. And where are the South, and the South-west? What was the South when we were young, is a long way short of what is considered to be the South now-a-days, and the West has travelled some thousands of miles farther off.

Four or five years since, Southern and South-western diseases and their treatment were only known and to be learned in the schools of Kentucky; but now the mantle has fallen from the shoulders of the venerable father of "sectional medicine," and has gone to Memphis and Augusta! How long will it be until it reaches Vera Cruz or Yucatan?

But seriously—what is meant by malarious diseases? Is Yellow fever a malarious disease, and is that confined to the South? Why it exists on Staten Island, a hundred miles north of Philadelphia, while we are writing this paragraph! Are intermittent and remittent fevers malarious? Not a summer passes that we do not have them in the alluvial districts which surround Philadelphia, of every grade, from mild tertian ague up to the most alarming remittent, and occasionally so severe that the patient dies in the first or second paroxysm. And yet we possess no opportunities for the study and treatment of malarious diseases! But, say some of the professors of physic south of Mason and Dixon's line, your malarious diseases are not like ours! Why not, we ask! Have *you* studied and treated the malarious diseases of the Middle States? If not, how do you make the comparison? Is there anything about southern and southwestern diseases that the observers of them have not told us? If there be, why hide they their light under a bushel? But there is nothing that has not been said a hundred times over. Descriptions and dissertations on southern diseases fill the pages of their journals, and make up the greater part of the books with which "the south and south-west" have favoured us; and if the physicians of those regions have observed well, and describe well, we know all about their diseases. Many of us, too, have seen them for ourselves—have bearded the lion in his den—and are consequently not obnoxious to the charge of ignorance bestowed so freely upon us.

Except those who go to Kentucky, the far greater part of the students, who travel North for medical instruction, visit the colleges of New York and Philadelphia, and we should like to know from our southern polemic, which of the professors of the Theory and Practice of Medicine, in the four large schools of those cities, are incompetent to teach a student how to treat malarious diseases? Every one of them has had to deal with yellow fever time and again, to say nothing of the milder forms of malarious fevers, which prevail more or less in the vicinity of those cities every year.

An esteemed correspondent, educated in the west, and familiar with the malarious diseases of the south-west previous to his entering the Navy, has given on another page *his experience* on this subject. He has not treated us with mere assertion, such as we have been combating; but has given facts, and substantial reasoning. Let us, instead of declamation, have facts. Where was the Southern editor himself educated, and where nearly all the able physicians of the South? In Northern Schools. Where were the major part of the physicians of the Army and Navy educated? In the same schools. Show us the instances in which the gentlemen in the public service who were educated in Northern schools proved incompetent to the discharge of their duties when stationed in the South, or those educated in the South or West were found wanting when performing duty in the North, and then we shall have something better to guide us than mere speculation; but until some such proof is afforded, we must be excused from subscribing to doctrines so repugnant to the dignity and usefulness of our science.

If the doctrine we are noticing be true, Congress ought to repeal the laws which authorize Surgeons to be appointed in the Army and Navy. The commanders of our ships should select their medical advisers from the native doctors of the climes wherever they may experience sickness; and our armies should be provided with physicians *pro re natâ* from among the Indians and Mexicans, and in every town or village through which they pass. Is the Editor of the Georgia Journal ready to head a petition to Congress for this reform?

The Southern Editor denies, that "the idea that a Student of Medicine must be taught his profession in the very locality in which he is destined to practise it, is now generally abandoned." "Such," he says, "may be the case at the North, but it is certainly not so at the South." We are sorry for it. Of all the wide world, the South—the southern portion of the United States—is the only place where such a disparaging doctrine is proclaimed. Why is it not as necessary to study medicine in New England for those who are destined to practise there, as it is for the Southern student to study in Augusta, New Orleans or Memphis? Does the Southern professor believe himself incapable of teaching his class the proper treatment of pneumonia, because it is less frequent and severe than in Boston or Portland? Are the physicians of London or Paris better able to treat convulsions than those of Georgia? Must every specialty be learned where the objects of it most abound,—or where is to be the limit of the rule prescribed?

"The great principles of the science are the same every where," he admits; "but," he adds, "their applications are as various as the races of man and the differences of climate." Ay, say we, and the various other modifying causes, as age, sex, habit, season, and atmosphere. It constitutes the great utility of the priciples of our science, that they are capable of being applied to the various conditions of the human system, induced by these modifying influences.

"Let Southern practitioners look into the numerous works upon the Practice of Medicine published in Europe or at the North, and he (they) will at once perceive," says the Editor, "that they contain but little of value in the management of our fevers." If not in these works, we should be glad to know where the desired information may be obtained? Dr. B. Rush Mitchell, however, has supplied the answer. The Navy Surgeons who were so eminently successful at Vera Cruz got their information from the very books which the Editor condemns, and were educated in Northern schools; and we hope it will not offend him to learn, that some of them were from Jefferson Medical College, and fresh from its halls.

In conclusion, we must say to our Southern friend, for so we still regard him, that we regret the tone of his remarks should have rendered it necessary for us to notice them. Nevertheless, if it is his purpose to maintain the position, that a student ought *not* "to seek for information wherever it can be best and most readily obtained;" and that a physician, however well instructed, *is unfit to practice except in the very locality where he has been taught*, we shall most cheerfully enter upon the discussion with him. But in doing so, let us not forget the proprieties of our profession, and impute wrong motives where arguments alone are the proper weapons.

